# Therapeutic horseback riding for at-risk adolescents in residential care

**DOI:** 10.1186/s13034-022-00523-5

**Published:** 2022-11-23

**Authors:** Shlomit Weiss-Dagan, Nofar Naim-Levi, Dorit Brafman

**Affiliations:** 1 School of Social Work, Bar-Ilan University, 52900 Ramat Gan, Israel; 2 School of Social Work, Ariel University, Kiryat Hamada 3, 40700 Ariel, Israel

**Keywords:** Therapeutic horseback riding, At-risk adolescents, Self-determination theory, Sense of competence, Autonomy

## Abstract

**Background:**

Over the past two decades, a large body of research has focused on the contribution of equine-assisted therapies to positive psychological changes in at-risk adolescents. The current study aimed to explore the subjective experiences of therapeutic horseback riding (THR, a type of equine-assisted therapy) among at-risk adolescents and examine how they describe the psychological benefits and the mechanisms of change of a THR intervention.

**Methods:**

This qualitative study focused on at-risk adolescents living in residential care. In-depth interviews were conducted with 19 adolescents.

**Results:**

Thematic analysis revealed three themes: (a) participants’ presentation, (b) the benefits of riding as a mechanism of change in the THR intervention, and (c) the benefits of the relationship with the horse as a mechanism of change in the THR intervention.

**Conclusions:**

The results of this study emphasize that the riding process and the relationship with the horse are the core mechanisms of THR interventions for at-risk adolescents and provide various psychological, behavioral, and relational benefits. Self-determination theory (SDT) is a relevant framework through which to explore at-risk adolescents’ subjective experiences of THR intervention and reveal its benefits for them.

## Introduction

Animal-assisted therapy (AAT) is defined as a “goal-directed intervention in which an animal that meets specific criteria is an integral part of the treatment process” [1, p. 264]. Equine-assisted therapy is a specific form of AAT. In general, the literature divides equine-assisted therapies into three subtypes: equine-facilitated psychotherapy (EFP), hippotherapy, and THR. Many conceptualizations and techniques are associated with EFP, and it may include a variety of unmounted or mounted activities conducted in groups or individually. Riding and horsemanship skills are the main focus of THR [2]. In the current study, we focused on at-risk adolescents in residential care who were taking part in THR. THR encompasses various elements of recreational horseback riding in addition to many other rehabilitative uses of the horse [3]. It is geared toward teaching riding and horsemanship while adapting the instruction to riders’ special needs [4]. Therapeutic riding programs usually treat individuals with physical and mental disabilities, serving as holistic therapeutic frameworks that seek to improve both physical and psychological aspects of their lives [5]. THR takes place in the natural surroundings of the stables and is designed to promote improvement in human physical, social, emotional, and/or cognitive function that cannot always be achieved by means of traditional treatment or rehabilitation [6].

At-risk adolescents are vulnerable to negative outcomes when they are exposed to adverse risk factors [7] such as family discord, maltreatment, low social competence, academic failure, and various socioeconomic factors, e.g., family income level, race, class, and neighborhood of residence [4, 8] may raise their risk levels. As a result, many of them engage in delinquency and experience emotional and behavioral difficulties. In this population, equine therapy has been demonstrated to be a viable alternative to conventional interventions. There has recently been a significant increase in studies on equine therapy with children and adolescents [2]. Empirical studies have detailed a variety of positive psychological outcomes of equine interventions with adolescents. These include enhanced self-control, self-image, life satisfaction [4], self-esteem, and self-efficacy as well as anxiety reduction and behavioral improvements [7–11]. Scholars have suggested that before future clinical trials to test THR’s effectiveness are designed, more robust theories regarding how THR influences outcomes are required [12]. The current study sought to address this need. It had two aims: first, to investigate at-risk adolescents’ subjective description of the benefits of THR intervention, and second, to shed light on THR’s mechanisms of change among at-risk adolescents. Mechanisms of change are the aspects of the intervention that facilitate and foster its benefits. It is important to explore adolescents’ subjective experiences of THR intervention to enhance our understanding of its benefits as they experience them. Their subjective experiences of THR can contribute to the design of future interventions, enhance their efficacy, and make it possible to tailor them to this population.

### The therapeutic role of THR and the horse as an agent of healing

Throughout history, humans have recognized the horse’s ability to heal minds and bodies. The horse has been used as a therapeutic agent since the days of ancient Greece [13]. Over time, the use of horses for therapy and rehabilitation gained popularity, and therapeutic riding programs proliferated worldwide, beginning in 1943 in Denmark, the birthplace of therapeutic riding [14], and continuing in England in the 1950s [15]. Since the 1990s, equine therapy has expanded rapidly in the United States and Europe [4].

One of the advantages of using an animal in a therapeutic setting is that it can help to reduce client arousal and socially mediate therapy sessions [16]. The fact that riding is a recreational activity mitigates the negative connotations associated with treatment or rehabilitation and may enhance both the rider’s engagement in therapeutic riding and the desired results [6]. Equine therapy specifically has been proposed as an alternative treatment for at-risk adolescents, so further investigation into its efficacy is warranted [7].

As herd animals, among which cooperation is very important and bonding between members exceptionally strong, horses are particularly suitable for therapeutic work. Herd dynamics are associated with life experiences such as friendship, rejection, cooperation, and injury and events such as birth and death [4]. The horse can provide advantages such as being non-judgmental [17] and serving as a metaphor for the rider’s internal world [18] as well as helping the rider build self-esteem, confidence, and mastery [19]. Some theorists suggest that the horse’s strength and large size provide therapeutic benefits, allowing riders to explore issues related to vulnerability, power, and control [20].

Human beings’ ability to ride horses allows them to understand and relate to the equine experience and deepen the therapeutic process through significant horse–human attachment [21]. The relationship with the horse facilitates the development of trust and love as well as a sense of moral responsibility toward another being [14].

### Benefits of equine-assisted therapy with at-risk adolescents

One of the challenges of therapy with at-risk youth is the difficulty of conducting verbal therapy. Children and adolescents may be unaware of their presenting problems or not perceive them as problematic and may therefore view therapy as unnecessary [22]. Traditional psychotherapy can comprise a “strange and unusual situation” for them [23]. Many alternative therapies, such as art therapy, adventure therapy, play therapy, and others involve non-verbal therapeutic interventions [24]. THR is one such therapy that offers a good option for at-risk youth.

A growing body of knowledge points to the psychological benefits of equine-assisted therapy. A meta-analysis of studies from 2005 to 2014 found that participation in an equine therapy program effectively increased the overall level of functioning of at-risk adolescents [7]. The studies we reviewed focused on EFP or hippotherapy in this population. Nonetheless, studies that focused on the riding aspect of horseback-riding interventions provide evidence of their benefits for at-risk adolescents. For example, in Burgon’s [8] qualitative study of at-risk adolescents’ experience of equine-assisted therapy programs, participants reported an increase in empathy as a result of the program. Another study of at-risk adolescents who participated in EFP, including therapeutic riding, found that having a 1400-pound horse respond to their commands in a non-threatening way provided participants with a sense of validation and contributed to their ability to regain control over their lives [25]. Several quantitative studies that examined the effects of EFP (including riding) among at-risk adolescents found psychological benefits such as increases in their trust, self-control, self-image, and general life satisfaction compared to a control group [4, 18]. A comparative study of the efficacy of equine-assisted therapies with at-risk children and adolescents also showed statistically significant improvements in social behavior [19].

In conclusion, the existing literature on equine-assisted activities, including riding, consistently emphasizes their beneficial effects on emotional well-being and social and interpersonal relationships. According to Burgon [8], equine-assisted activities may be considered beneficial interventions for at-risk adolescents, even without a great deal of psychotherapeutic content. In light of the empirical findings, this claim raises the question of what aspects of THR intervention comprise the mechanisms of change that benefit at-risk adolescents.

### Mechanisms of change of THR intervention

The literature that addresses the mechanisms of change of THR intervention is scant and none of it focuses on at-risk adolescents. One study that explored THR’s mechanisms of change found that children with disabilities who took part in THR had experiences that led them to have more positive views of themselves [12]. The THR intervention provided them with agency that enabled them to learn to move, succeed, connect with the horses, and adapt to new circumstances while responding appropriately. These four levels of learning served as the mechanisms of change that expanded their self-concept through THR intervention. In another study that examined the same population, Martin et al. [26] revealed additional aspects of THR’s mechanisms of change, such as building riders’ capabilities and strengths and enhancing their enjoyment. In both studies, Martin and his colleagues emphasize the importance of the rider’s role as an active agent. They mention self-determination theory (SDT) [27] and the need to fulfill three basic needs—competence, autonomy, and relatedness—that enable children to become engaged and active participants in treatment. SDT is a classical theory of development and human motivation [28] that views human behavior as growth-oriented and proactive [29]. According to SDT, the fulfillment of the three basic needs of competence, autonomy, and relatedness supports children’s inner motivation and resources and facilitates change [30].

Another qualitative study [31] explored the significance of participation in a THR intervention among young people with physical disabilities. Using causal agency theory [32] as a framework, the researchers interpreted participants’ experiences. Two of the themes they observed were “horseback riding is not a sport but a therapeutic activity” and “relationships with peers and instructors facilitate opportunities for self-determination” [27]. The adolescent participants’ sense of independence and autonomy increased as a result of their relationships with the horses. Developing a sense of independence became possible when participants felt safe riding, but this possibility was limited when they received too much help from instructors [31]**.** That is, a positive connection with the instructors and horses was the mechanism that facilitated autonomy, comfort, and enjoyment.

To the best of our knowledge, no studies have examined THR’s mechanisms of change in at-risk adolescents, a population with a unique set of difficulties and challenges. In the studies summarized above on THR in children and adolescents with disabilities, the importance of control and agency is emphasized. The present study was conducted to shed light on the subjective perception of benefits and the mechanisms of change of a THR intervention among at-risk adolescents.

## Method

### The participants

The research participants were 19 adolescents (aged 14 to 19), all of whom were in residential care and taking part in a THR program. They comprised two groups on the same continuum of risk. The first included adolescents from religious residential care who were not integrated into schools in the community (*n* = 11). The members of the second group (*n* = 8) had been involved in the past with drug abuse, theft, and violence, and came from juvenile justice facilities that provided stricter residential care and were supervised by the Israeli Ministry of Social Affairs and Social Services. Previous attempts to treat them had been unsuccessful. Residential care is part of the rehabilitation process and is sometimes a condition for avoiding incarceration. The adolescents had been placed in these institutions either by court order or by child protection social workers. Many of them had serious difficulties, vulnerabilities, and complex family situations, and some had suffered from physical or sexual abuse. The purpose of these institutions is to provide a safe space with clear boundaries for these young people’s growth and education [33]. The adolescents from the juvenile justice facilities are at the other end of the continuum of risk. Most of them have criminal records and have endured harsher life experiences such as exclusion, neglect, and abuse. These two groups take part in the same THR intervention and come weekly to the farm for therapeutic riding.

Both facilities are for boys only. Seven of the participants were aged 14 to 16. Most were 17 years old (*n* = 10) and another two were 18 to 19 years old. Three reported that their parents were divorced, one shared that his father had passed away, and two had been raised by single mothers. Five were immigrants. Their periods of residence at the facilities ranged from 4 months to 4 years. Six had previous horseback riding experience. All the participants had experienced frequent transitions between schools and residential care facilities.

### Reflexivity

In describing the data collection process, we cannot ignore its reflective element. Both first authors are middle-class, academic, adult women and social workers. These aspects of our identity were known to the participants. We introduced ourselves as social work researchers. Both interviewers felt that the fact that they were social workers had considerable impact on the participants. Since their past encounters with social workers had taken place in the framework of their placement in residential care or interactions with an authoritarian probation officer, for many of them the profession had negative associations. From an intersubjective perspective, this knowledge affected our initial interactions with the participants, and we took special care to create a pleasant, non-judgmental atmosphere to counteract any possible effects of these associations. Taking into account the power dynamics related to age, socioeconomic status, and education that existed between us, we took a stance that acknowledged the valuable knowledge the participants had acquired through their life experiences. We endeavored to convey the message that their knowledge was valid and no less important than ours since we were genuinely curious about it and sought to learn from their insights.

### Research setting

The study was conducted at the TRCI (Therapeutic Riding and Canine Center in Israel), a THR farm. The adolescents’ routine at the farm included 6 weekly hours of THR sessions. During the sessions, participants rode the horses freely in an enclosed area or the area surrounding the farm; took riding lessons in which they were taught ridings techniques and learned how body language and attitude affect horses; cared for the horses after riding, including washing, brushing, and feeding them; and took part in group sessions on the riding experience and the relationship with the horse. Horse care is an essential part of the therapeutic process and includes a gradual process of increasing adolescents’ autonomy in taking care of the horses. At the beginning of the process, the riding instructors supervise the horse care. Sometimes at this stage, adolescents may become frustrated and impatient with the horses, but as their relationships with them grow stronger, they become capable of placing the animals’ needs before their own. When the instructors feel they can trust them, they are gradually allowed a greater measure of autonomy in horse care. Part of the intervention’s aim is to provide a stable, predictable setting with clear order and boundaries. In this regard, both riding techniques and horse care have clear rules.

Due to the adolescents’ behavioral patterns and social exclusion, the purpose of the THR intervention, as defined by the residential care facilities, is to promote psychological, social, and behavioral change that will enable them to integrate into society through normative channels such as military service (compulsory in Israel), employment, and higher education. THR is a unique type of intervention used in residential care. The special characteristics of this kind of intervention are the natural, relaxing space of the farm, the challenge of the physical activity involved, and the relationship with the horse. In other residential care interventions, these unique elements are absent.

### Data collection methods

The study was approved by the ethics committee of the first two authors’ university. The residential care facility obtained the parents’ permission for the adolescents to take part in the study. We invited all 21 adolescents who were taking part in the THR intervention to participate. Two of them refused. Nineteen agreed voluntarily to participate, and each signed an informed consent form. Pseudonyms are used here, and other identifying information was changed to preserve participants’ anonymity.

We began the data collection by presenting the aims of the study to the adolescents’ riding instructors at the farm and their counselors at the residential care facility. We did so based on the understanding that collaboration between adolescents and adult researchers should be supported by the institutions involved [34], especially when it concerns at-risk adolescents who have trust issues.

The data collection methods included in-depth individual interviews aimed at shedding light on the participants’ subjective experiences. The study consisted of 19 semi-structured interviews conducted by the first two authors with 19 adolescents over 2 months in 2019. Some took place at the farm and some at the residential care facility. The interviews lasted 40 to 80 min each. At the beginning of the interviews, we explained to the participants that we were exploring their experiences at the farm and learning from their knowledge. First, we asked them to tell us about themselves. Next, our main request was “Tell me about your experience at the therapeutic riding farm.” Then we asked them to share a significant experience they had had at the farm. Furthermore, we asked them to tell us how their experiences at the farm and their relationships with the horses and riding instructors subjectively influenced them and affected them outside the farm context. Finally, we asked them what changes created by THR they could identify in their lives following their experience on the farm.

### Data analysis methods

The thematic analysis process included several phases, and we followed Braun and Clarke’s [35] phases. In the first phase, we conducted an inductive analysis. Each of the authors read each interview separately and gave each paragraph a preliminary title. Next, in the second phase, each of the authors compared inter-interviewee and intra-interviewee responses and identified dominant codes. In this way, 24 distinct codes were generated. To increase credibility, we looked for commonalities in the codes and definitions. Codes that were present in both lists were included in a separate list. Next, we reviewed all the data again and each of us performed an initial deconstruction of the texts and sorted them into general themes that arose from the data. The themes facilitated the comparison and identification of shared meanings and patterns that emerged from the data relevant to each one. Throughout this process, we utilized three themes: adolescents’ background, the benefits of riding as a mechanism of change in THR, and the benefits of the relationship with the horse as a mechanism of change in THR.

We then checked whether the themes worked with the coded extracts and the entire data set and created a thematic map. Next, we defined the subthemes using the previous list of codes and sorted the codes into subthemes [36]. A validity assessment of the final dataset was produced by double-checking to ensure the codes that referred to quotations were applied consistently across all interviews.

## Findings

### The participants’ presentation of their past and current difficulties

We begin by introducing the subjective self-presentation of the participants and their personal histories. Since the adolescents in the study had experienced social exclusion throughout their lives, we sought to make their voices heard and enable them to define and introduce themselves in their own words. We focus here on the main difficulties shared by the participants. Sixteen participants reported histories of frequently moving between schools and residential care facilities. All of the religious adolescents had experienced social exclusion and entered residential care at their parents’ request or following the recommendations of members of their extended families, friends, or community members after not fitting into other schools. The following excerpt provides examples of such experiences:

*I was not accepted in the religious community, and I was not accepted at any regular religious boarding school. So I went to a residential care facility for boys with attention and concentration difficulties, but they expelled me after about four months because I was messing around. I didn’t like being there. Then I was at home for about two months until I arrived at this residential care facility.* (Joseph)

The adolescents from juvenile justice facilities also experienced social exclusion, as the following excerpt demonstrates:

*I was in some other boarding school for a year and then I left, and I had no educational framework. I tried to get accepted to another residential care facility and didn't succeed, so I stayed at home for more than six months. During this time, a police case was opened against me for assault, and that’s how I ended up here.* (Benny)

Eighteen of the adolescents shared a lack of a sense of belonging and a strong sense of alienation. These feelings stemmed from their experiences of being from immigrant families, failing to integrate into schools, moving frequently, lacking acceptance in religious society, feeling different, and more:

*I wasn’t like the other kids there. In terms of behavior . . . how I dressed, my appearance … They liked to go and play … with their friends at that youth center, and I liked to smoke and drink and go out with kids from another settlement.* (Billy)

Some of the interviewees spoke of abusing drugs and alcohol (eight participants) and engaging in theft and violence. Seven participants mentioned their age-inappropriate responsibilities or experiences:

*From age eight … I worked in gardening. I would clean stairwells [in apartment buildings]. I didn’t want to ask my parents for anything. I didn’t want to need anyone or ask for money. At age thirteen, I would hang around with people aged seventeen, eighteen, twenty-nine, or thirty. Let’s just say that a thirteen-year-old kid shouldn’t have been hanging around with them.* (Eli)

Five participants described the negative influence of their neighborhoods. The following excerpt is an example of this type of experience:

*I can tell you that in my neighborhood, everyone who was with me, people in their twenties and thirties, they’re all in prison now. It’s the neighborhood … I went through things there that I shouldn’t have gone through . . .* (Shon)

The above excerpts highlight a variety of difficulties experienced by the study participants, who revealed a series of past and present struggles and challenges such as frequent moves between schools, a lack of a sense of belonging, social exclusion, criminal offenses, drug abuse, and age-inappropriate responsibilities. The participants’ past challenges can shed light on THR’s contribution to their present ability to cope.

### The benefits of riding as a mechanism of change in THR

Sixteen of the adolescents described the THR as a positive experience. They experienced excitement and expectation before coming to the farm. They spoke about enjoying the experience, which encouraged them to stay despite the obstacles and challenges they faced at the residential care facility. They emphasized riding and horse care as enjoyable activities. Eight referred to the benefits of riding as a way to release aggression and energy. The riding itself had a relaxing effect on them. This effect is an important contribution of THR, especially for at-risk adolescents, who may exhibit physical and verbal violence at the beginning of the therapeutic process. They felt that riding on a given day had a positive impact on their functioning for the rest of that day and even the next day. This means that the riding itself was a mechanism of change in terms of relaxing and functioning better at school. Five of the participants described the calming effect of the farm’s natural surroundings and open space. The following quote relates to these aspects of the intervention’s effect:

*It’s a little hard to see the impact of riding and understand what affects me, but I do understand and know it only does me good. It makes me more energetic at school and it’s a place to come and let go. It’s not like you get up in the morning and go study. You get up, come to the farm, to a place that’s more comfortable, somehow easier for me and more … I don’t know how to explain … and then I go back to the residential care and feel much better there.* (Moni)

Participants also spoke of their efforts to cope with their low frustration thresholds while riding. One of them explained:

*Sometimes it’s a bummer here, sometimes not. But I have to cope with it if I want to stay here. When you don’t succeed at something, you need to work collaboratively. I have a low frustration threshold, so I get upset quickly, and then I have to deal with it . . . Here I have to make an effort. Because I like it. Yes, I like it, even if there are frustrations …* (Abe)

Through riding, the adolescents underwent a process that required perseverance and long-term investment. At some point, they began to see the fruits of their hard work; their riding techniques improved, and they gained the horses’ trust. This persistent investment in riding was beneficial since it influenced them to invest more consistently in other activities (e.g., other sports) and in their studies. One participant told us the following:

*After the farm, I have a math lesson and the day is much harder, but even though it ends in the evening, it’s an easier day for me. That’s what I feel. It’s good for me. It helps me let things out, you know, not sitting all day … I don’t know if I could do it.* (Moni)

The adolescents’ persistence was an additional benefit of the riding process and helped them learn how to cope with failures and enjoy success, as one of the participants explained:

*The road to success involves falling several times and either succeeding or falling. When you’re with horses you have thousands of failures every day and once in a while a small success. Most of the time, it’s failures and occasionally you have one of those small successes and it fills you with happiness.* (Joni)

Fourteen of the interviewees mentioned their improved sense of competence following their THR experience. They attributed this improvement to having learned how to ride successfully and how to be patient, having undergone a long process that required perseverance, and having made progress and acquired skills in riding, the fruits of their labor. Participants also spoke of the experience of not giving up on themselves. In addition, they mentioned hard work, meeting riding challenges, and competition as meaningful factors that significantly impacted their sense of competence. The following excerpt shows how competition boosts confidence:

*When I ride the horse and it’s hard for me to do what I need to do, I struggle with myself and, in the end, I succeed and overcome the difficulty. … I feel that I learn something new every time I ride.* (Dan)

Six participants emphasized their perception of autonomy and free choice in riding. One of them described the benefit of the independence and autonomy he gained by developing an inner motivation to ride:

*If you don’t come [to THR] for a while, they will kick you off of the farm. But even here [at the farm], let’s say I have a choice. If I don’t want to ride today, I won’t ride. There was a time when I didn’t want to ride, and then I saw how everyone was progressing, and I said well, I’ll try. Slowly, slowly you evolve and progress. You know? This is what I have wanted all my life. I wanted my independence.* (Avraham)

In short, the participants’ subjective experiences revealed different benefits of the THR intervention in terms of their functioning, coping, and mental states. From the participants’ subjective experiences, it emerged that the main mechanism of change of the THR intervention was evident in the riding experiences.

### The benefits of the relationship with the horse as a mechanism of change

Alongside the riding itself, the relationship with the horse was a dominant mechanism of change. Some participants reported that their relationships with the horses and the processes they underwent increased their sense of competence. One spoke of the horse’s strength and its contribution to the confidence and ability involved in horseback riding:

*Sure, once you get on a horse and learn how to control it you realize, wow! The first time you get on a horse, you don’t understand how people do things with it … it increases your self-confidence and sense of self-worth … you see that you’re controlling such a powerful animal and the more you learn, the more you understand its power and its gentleness. You learn about yourself.* (Joni)

The relationship with the horse also helped some of the adolescents to face and overcome their fears. One described his inner struggle with his fear of the horse and the satisfaction of mastering it. Another spoke of overcoming the trauma he had experienced after falling off the horse and how this experience had contributed to his sense of competence:

*At the farm, I fell. A horse threw me. I went to the hospital, got X-rays. Then I was afraid to mount the horse. I didn’t want to do it anymore. So, for about six months, I … got on for a few minutes, my hands shaking, and said “no, not interested,” that’s it. Until I got to my horse, Donatello. I reached out to him and trusted him. I don’t know why. I had a connection with this horse. In the end, he helped me . . . I don’t know how. Today … with the help of the horse I . . . [can] go in, hug him, kiss him … I do everything alone. I don’t need anyone to be with me …*

In the last two excerpts, it is apparent that the participants’ sense of competence and ability to control the horses increased thanks to their relationships with the horses. These relationships helped them gain a sense of control over the horses and overcome their fears and failures.

The next excerpt shows specifically how the relationship with the horse affected the need for autonomy and demonstrates how it clarified for participants the boundaries of their autonomy and the meaning of autonomy in a relational, intersubjective context.

*It’s … easiest to give up and say it doesn’t depend on me, it depends on the horse, the horse decides. And what it mainly taught me is that many things depend on me too. We work together … I started looking for the gray areas in riding … like the difference between pulling too hard or too weakly to get the [desired] action and doing it exactly right …* (Nathan)

A sense of freedom and choice are part of autonomy. In the context of the relationship with the horse, some of the participants discovered the boundaries of their own autonomy and became more sensitive to their horses. The participant quoted in the following excerpt described the horse as an extrinsic entity that had the power to decide and take control when he attempted the jumping exercise with it. As this excerpt reveals, when the rider relinquishes control, the horse begins to cooperate, and the resulting mutual trust can lead to success.

*Once, there was a competition that I trained for a lot and … I was supposed to do jumps … I was the only one on the team who did jumps, and I was very excited. And I was last. Everyone was finished and then I came in with the horse … I was very, very stressed and I had done these obstacles with the horse dozens of times. Because I was stressed … I completely ignored the horse and was only focused on myself and … I got to the obstacle and the horse didn’t jump and I was so frustrated and started cursing … I blamed the horse and said, “he’s having a bad day” and … then a week later I succeeded, and the horse was the same horse, and everything was the same, and I realized how much my stress had affected those subtleties.* (Dave)

Autonomy is a universal need. Through their relationships with the horses, our adolescent participants learned to sublimate their need for autonomy. Doing so helped them to be more sensitive to the horses, cooperate with them, and progress.

Gaining trust within a relationship was another important benefit of the relationship with the horse. We identified the central, fundamental need for trust when the participants spoke about their meaningful relationships with the horses (10 participants). One participant talked about the different ways in which he allowed himself to connect with his horse, despite his difficulties in connecting with people:

*It annoyed me that they took [my horse] Shrek… and didn’t tell me ahead of time. I was suddenly informed two weeks later, as if they had just taken him. It was very annoying. It’s your horse, yours. So, with the new horse, I was devoted, I gave him all my love, all my warmth. But today I know that even if I leave someone, even though I don’t know what will happen, I know that … I’ll soon take everything he says with a grain of salt. I’m very emotionally closed in that way. I also have a hard time with love.* (Eli)

Another participant spoke of the process of relinquishing control after establishing a connection with the horse and learning to trust it:

*I think the most significant thing … was … the jumps. There … you do something beyond [the usual]... it requires a lot of precision and … trust in the horse and suddenly you’re in situations … where you know that you’re not in control … that the work here is both the horse’s and yours … and at the moment of the jump you let go of everything and let her do the work. I learned to trust.* (Nathan)

Some participants talked about nonverbal communication with the horses, the sense of responsibility for them, and the mutual understanding between them and the horses. In this regard, Guy explained the following:

*I felt as if [the horse] felt safe with me because she was insecure and used to being scared and hadn’t found a leader and was part of the herd … even today she loves me when I come to say hello. What I learned is that she’s an animal … she can’t talk … I have to know when something hurts her, when she doesn’t want to do something, when she feels good, what she likes.* (Guy)

Seven of the participants talked about the horse as a symbolic projection of a human being, as in the following excerpt:

*Yes, sometimes … like people, horses can get up on the wrong side … and then you go to the horse and immediately notice that he’s a little sad or nervous. And then you need to know what to do. And how to communicate with it and how to calm it down if necessary … or how to say to it, OK, get up, wake yourself up.* (Doron)

Seven participants also felt that the relationship with the horse made them more sensitive to others, as this excerpt demonstrates:

*Riding … is working together, working with the animal, understanding it and being considerate of it. For example, it’s having a hard day and you have to understand it and say, OK, don’t make such an effort. And you’ll still do it. And that is teamwork. You have to take care of the animal after you ride, wash it. It’s like a part of you … The work with the horse helps you show more emotions, notice things. The horse may be limping a bit or something like that and sometimes you can’t see it, but you understand it doesn’t feel well. You learn to look from the outside, to check all kinds of things to make sure they’re OK. It taught me a great deal … to invest in relationships …* (Moses)

The participant felt that he could give something to the horse, teach it, lead it, and have someone to care for, love, and nurture. These are all meaningful relationship experiences for participants. The relationship with the horse was significant in terms of participants gaining competence and balancing autonomy and trust.

Notably, only three participants described their experiences as negative. Each one of them had a different reason for feeling this way about the THR, for example, a lack of connection with the horse or a lack of interest in the riding style taught at the farm. The following quote provides an example of such sentiments and attitudes:

*I never connected with this animal or the one I was forced to ride from the beginning. I didn’t want to ride. Over time it changed a bit. I started to be less against it, but I never liked it. There were some lessons when I wouldn’t go up to ride. Instead, I would just work on the farm.*

For all of the participants except these three, THR was a meaningful and constituent experience, as the findings demonstrate (Table 1).

**Table 1:** Theme and sub-themes: cross-case analysis

1. Benefits of riding as a mechanism of change in THR	
1.1 Relaxing and functioning better	*It makes me more energetic at school and it’s a place to come and let go. It’s not like you get up in the morning and go study. You get up, come to the farm, to a place that’s more comfortable, somehow easier for me…*
1.2 Balancing and coping better with frustration	*I have a low frustration threshold, so I get upset quickly, and then I have to deal with it... Here I have to make an effort. Because I like it. Yes, I like it, even if there are frustrations..*
1.3 Persevering and acting consistently in other activities	*After the farm, I have a math lesson and the day is much harder, but even though it ends in the evening, it’s an easier day for me. That’s what I feel. It’s good for me. It helps me let things out, you know, not sitting all day … I don’t know if I could do it*
1.4 Coping with failures and enjoying success	*The road to success involves falling several times and either succeeding or falling. When you’re with horses you have thousands of failures every day and once in a while a small success. Most of the time, it’s failures and occasionally you have one of those small successes and it fills you with happiness*
1.5 Gaining a sense of competence by meeting riding challenges	*When I ride the horse and it’s hard for me to do what I need to do, I struggle with myself and, in the end, I succeed and overcome the difficulty. …*
1.6 Gaining autonomy and free choice in riding	*…let’s say I have a choice. If I don’t want to ride today, I won’t ride. There was a time when I didn’t want to ride, and then I saw how everyone was progressing, and I said well, I’ll try. Slowly, slowly you evolve and progress*
2. Benefits of the relationship with the horse as a mechanism of change in THR	
2.1 Increased confidence thanks to controlling a powerful horse	*…it increases your self-confidence and sense of self-worth... you see that you’re controlling such a powerful animal*…
2.2 The ability to face and overcome their fears	*At the farm, I fell. A horse threw me… Then I was afraid to mount the horse… Until I got to my horse, Donatello. I reached out to him and trusted him. I don’t know why. I had a connection with this horse. In the end, he helped me... Today... I do everything alone. I don’t need anyone to be with me*.
2.3 Autonomy, mutual and internal locus of control within the horse–adolescent relationship	*It’s... easiest to give up and say it doesn’t depend on me, it depends on the horse, the horse decides. And what it mainly taught me is that many things depend on me too. We work together...like the difference between pulling too hard or too weakly to get the [desired] action and doing it exactly right..*
2.4 The boundaries of autonomy within the horse–adolescent relationship	*Because I was stressed... I completely ignored the horse and was only focused on myself and... I got to the obstacle and the horse didn’t jump and I was so frustrated and started cursing... I really started getting upset and then they told me to get off the horse*
2.5 Connecting with the horse, despite difficulties in connecting with people	*It annoyed me that they took Shrek [my horse]... and didn’t tell me ahead of time… So, with the new horse, I was devoted, I gave him all my love, all my warmth. But today I know that even if I leave someone, even though I don’t know what will happen, I know that... I’ll soon take everything he says with a grain of salt. I'm very emotionally closed in that way. I also have a hard time with love*
2.6 Learning to trust the horse	*… the jumps…it requires a lot of precision and... trust in the horse and suddenly you’re in situations... where you know that you’re not in control...and at the moment of the jump you let go of everything and let her do the work. I learned to trust*
2.7 Nonverbal communication with the horse, responsibility, and mutual understanding	*she’s an animal... she can’t talk... I have to know when something hurts her, when she doesn’t want to do something, when she feels good, what she likes*
2.8 The horse as a symbolic projection of a human being	*… like people, horses can get up on the wrong side... and then you go to the horse and immediately notice that he’s a little sad or nervous. And how to communicate with it and how to calm it down if necessary*
2.9 Increased sensitivity to others thanks to the relationship with the horse	*You have to take care of the animal after you ride, wash it. It’s like a part of you... The work with the horse helps you show more emotions, notice things*

## Discussion

In this study, we aimed to deepen our understanding of at-risk adolescents’ subjective experiences of the benefits of a THR intervention’s mechanisms of change. As the adolescents described in their own words, their backgrounds are fraught with difficulties and challenges in different areas of their lives. The participants’ negative life experiences—delinquency, alcohol and drug abuse, and frequent moving between schools—all rooted in their social contexts, neighborhoods, and socioeconomic status. Due to these background factors, many of the participants did not have an equal opportunity to achieve success. Thus, THR provided most of them with a unique opportunity to flourish for the first time. The THR farm is, in this sense, a space in which adolescents can experience new roles, responsibilities, and physical and relational challenges. The moratorium of the THR farm provided the adolescents with an opportunity to experience new internal and external experiences important for their growth and rehabilitation.

The theme of the benefits of the mechanisms of change of the THR as described by the participants reveals different aspects of these subjective experiences. Some of these aspects relate to the effect of the interrelated mental and physical benefits of riding, such as the relaxation effect, the opportunity to release aggression, and improvement in scholastic achievements. Another benefit of riding mechanisms is related to the ability to persevere in other activities.

The need for competence is the need to feel effective, to feel one has an impact on one’s environment and is achieving positive outcomes [43]. One may satisfy this need by actively seeking challenges that expand one’s skills [37]. The riding experience is instrumental in this process. Our findings demonstrate the contribution of the riding experience and meeting its challenges to the participants’ sense of competence. Studies have shown that ongoing feelings of competence are important for optimal functioning and well-being [38]. Sense of competence is significant for these adolescents, especially considering their past negative experiences and failures.

The need for autonomy is satisfied when an individual experiences choice and volition in his actions and perceives himself as the origin of his actions. Autonomous actions are those that are self-endorsed and congruent with one’s values and interests [39]. The need for autonomy is part of the maturation process. According to Muuss [40], autonomy is one of the most vital components of identity development in adolescence. Furthermore, Blos [41] defines autonomy as a crucial part of the second individuation process of adolescence. Even when a degree of dependence on another person exists, freedom of choice is a key component of autonomy [42]. Autonomy and independence are important for human growth, well-being, and motivation [27]. Riding provides the benefit of free choice and control through a learning process, as the participants described.

The theme of the mechanism of change of the relationship with the horse is another unique aspect of THR intervention. The new positive relationship experiences with the horse include trust, mutual give-and-take, and sensitivity, which can carry over into other relationships in participants’ lives. Like all relationships, the rider–horse relationship is complex. The correct match between horse and rider, which is based on physical features and personality traits, is essential for successful therapy or rehabilitation [3, 6]. But when there is a good match, the relationship with the horse is a crucial factor in facilitating positive psychological and behavioral change in at-risk adolescents. This relationship with the horses helped some of the adolescents to regain trust. As mentioned in the findings section, three of the participants described their experiences as negative, and this may be explained by the lack of connection to the horses revealed in their statements. One of these three participants also reported that he felt disconnected from the residential care staff, supporting our explanation that without relatedness, there is no positive change. In our interviews, we asked the adolescents to tell us about their experiences at the therapeutic riding farm and were surprised by the dominant role their relationships with the horses played in their lives. It was clear that these relationships comprised an important mechanism of change.

In their relationships with the horses, the participants were able to gain an internal locus of control, confidence, and competence and clarify the boundaries of their own autonomy. The relationships with the horses made them more sensitive to the animals and served as mechanisms of change. The three basic needs as defined by SDT are relevant to these findings and can be used as an explanatory framework. SDT frames optimal human development as the interaction between humans who are striving for growth and their social environments, in which basic psychological needs (competence, autonomy, and relatedness) are either supported or thwarted [42]. SDT suggests that to be satisfied and thrive, people require these three specific basic needs to be fulfilled [43]. Our findings show that these three needs can be satisfied through THR intervention with at-risk adolescents. Sense of competence and autonomy are benefits of both mechanisms: the riding process and relationship with the horse.

In the context of THR and through their relationships with the horses, the participants gained complex insights into autonomy in terms of both their need for it and its limitations. Their relationships with the horses were instrumental in supporting their maturation processes and teaching them to see beyond themselves and their own needs. The THR experience taught them that they could not force their will on the horses and that horses do not necessarily cooperate. They learned that when they relinquish control, the horse begins to trust them, with the result that their trust in the horse increases and they make progress. Elkind [44] describes the gradual transformation of adolescents from being primarily concerned with themselves to recognizing the difference between their preoccupations and the interests of others. This insight is crucial to establishing mutual rather than self-interested interpersonal relationships [44].

According to SDT, relatedness is the need to feel connected to others and experience a sense of belonging. This need is satisfied when one develops close and intimate relationships with others [37]. Previous studies have emphasized the importance of the therapeutic relationships between adolescents and care workers and pointed to them as important in bringing about behavioral change in adolescents in residential treatment and/or their families [45]. In the case of THR with at-risk adolescents, our findings indicate that the relatedness between adolescents and horses is a crucial aspect of the intervention and can be productive and facilitate corrective experiences at several levels. Relatedness is a benefit of THR intervention but also a mechanism that enables competence, autonomy, and trust within the relationship with the horse.

Participants in both groups described a desire for autonomy in the form of freedom of choice, independence, and control. They also manifested a similar sense of competence as a result of experiencing success and overcoming challenges in riding. The main difference between the two groups was that the participants from juvenile justice facilities described their experience at the farm with more emotional language and emphasized their relationships with the horses. They attributed more human qualities, behavior, and feelings to the horses and projected their inner feelings onto them. Yet the descriptions of the effect of participants’ relationships with the horses on their lives, their sensitivity, and their ability to develop closer relationships with others were similar among participants from both groups.

Studies that focus on THR’s mechanisms of change among children with disabilities emphasize autonomy and agency as the most vital aspects of THR interventions. Our findings emphasize the importance of the riding process and the rider’s relationship with the horse as the main beneficial mechanisms of change of THR intervention for at-risk adolescents. Their specific challenges and life experiences, especially their past failures and lack of a sense of belonging and social exclusion, damaged their trust and self-confidence. Riding and the relationships with the horses give them a unique opportunity to begin anew and benefit from a corrective experience.

## Limitations

In the current study, we used thematic analysis to analyze our findings. This method has limitations; breaking the text down into codes and themes may affect the meaning of the text as a whole, and the constant comparison of texts may affect the depth of understanding of individuals’ states of mind. In addition, the results relate to adolescent boys in residential treatment, so the scope of the findings’ generalization is limited and they may not apply to at-risk girls or other equine-assisted therapies or treatment environments. Another limitation is that we did not compare the THR experience of the adolescents in this study to the experiences of other populations, so we cannot be certain that the findings apply exclusively to at-risk adolescents. Moreover, we did not employ a longitudinal study design, so we do not know the effect of the THR intervention in the period after we conducted the interviews. Future studies can explore appropriate courses of treatment and determine whether THR interventions reduce verbal or physical violence and whether the treatment results are durable.

## Conclusions and implications

The interviews revealed the key mechanisms of the riding process and the relationship with the horse as instigators of beneficial change. THR interventions with adolescents can be beneficial and fulfill the three basic needs of competence, autonomy, and relatedness. The fulfillment of these needs can increase the motivation for treatment [46] and is associated with psychological well-being [47]. THR with an emphasis on riding and the adolescent–horse relationship can help at-risk adolescents make positive psychological and relational changes. More research around SDT and equine-assisted therapy is needed and can give us additional insights into the mechanisms of change of THR interventions.

## Data Availability

Not applicable.

## Appendix

1. The informed consent form signed by the participants was in Hebrew.
